# Malaria transmission through the mosquito requires the function of the OMD protein

**DOI:** 10.1371/journal.pone.0222226

**Published:** 2019-09-25

**Authors:** Chiara Currà, Jessica Kehrer, Leandro Lemgruber, Patricia A. G. C. Silva, Lucia Bertuccini, Fabiana Superti, Tomasino Pace, Marta Ponzi, Friedrich Frischknecht, Inga Siden-Kiamos, Gunnar R. Mair

**Affiliations:** 1 Institute of Molecular Biology and Biotechnology, FORTH, Heraklion, Greece; 2 Integrative Parasitology, Center for Infectious Diseases, University of Heidelberg Medical School, Heidelberg, Germany; 3 Instituto Medicina Molecular, Lisbon, Portugal; 4 Core Facilities, National Institute of Health, Rome, Italy; 5 National Center for Innovative Technologies in Public Health, National Institute of Health, Rome, Italy; 6 Department of Infectious Diseases, National Institute of Health, Rome, Italy; 7 Iowa State University, Biomedical Sciences, Ames, Iowa, United States of America; Université Pierre et Marie Curie, FRANCE

## Abstract

Ookinetes, one of the motile and invasive forms of the malaria parasite, rely on gliding motility in order to establish an infection in the mosquito host. Here we characterize the protein PBANKA_0407300 which is conserved in the *Plasmodium* genus but lacks significant similarity to proteins of other eukaryotes. It is expressed in gametocytes and throughout the invasive mosquito stages of *P*. *berghei*, but is absent from asexual blood stages. Mutants lacking the protein developed morphologically normal ookinetes that were devoid of productive motility although some stretching movement could be detected. We therefore named the protein Ookinete Motility Deficient (OMD). Several key factors known to be involved in motility however were normally expressed and localized in the mutant. Importantly, the mutant failed to establish an infection in the mosquito which resulted in a total malaria transmission blockade.

## Introduction

Transmission of malaria parasites to the mosquito vector entails a complex series of events during the first 24 hours following a blood meal. Maturation of blood stage gametocytes into gametes takes place in the mosquito midgut immediately after uptake of the blood meal. This is followed by egress of gametes from the host erythrocyte, fertilization and formation of a motile ookinete from the round zygote. Motility of the ookinete is essential for the establishment of an infection in the mosquito, as it has to escape from the hostile blood meal environment by passing through the peritrophic membrane and traversing the midgut epithelial cells to reach the distal side of the epithelium where it will establish an oocyst.

Ookinete migration through the midgut epithelium is critical for continuation of the life cycle. A functioning acto-myosin motor is absolutely required, to provide power for gliding motility (for a review see [1]). The acto-myosin motor also depends on associated proteins and together they constitute the so called glideosome; these include a myosin light chain named MTIP and the gliding associated proteins GAP45, GAP50 and GAP40 [1] as well as the actin-binding protein glideosome-associated connector [2]. Secretion of proteins from specialized secretory organelles, the micronemes, is also essential and these function as adhesins linking the acto-myosin motor to the extracellular substrate. In ookinetes the micronemal protein CTRP (circumsporozoite- and TRAP-related protein) is necessary for productive motility, and mutants lacking the protein are completely blocked in formation of oocysts [3–5]. Other micronemal proteins are SOAP (secreted ookinete adhesive protein) and WARP (von Willebrand factor A domain-related protein), although they do not have a direct role in motility [6,7]. Furthermore, ookinete motility is regulated by kinases [8–10] and a phosphatase with kelch-like domains has also been implicated [11].

Here we explore the function of the protein PBANKA_0407300 from the rodent malaria parasite *P*. *berghei*. The protein is conserved within the *Plasmodium* genus but without significant similarity to proteins of other eukaryotes. Absent in blood stage asexual parasites the protein is first expressed in the gametocyte, however it plays no role in fertilization or ookinete formation. Instead, the protein is essential for gliding motility and thus named Ookinete Motility Deficient; *omd* null mutants fail to parasitize the mosquito vector resulting in an absolute malaria transmission blockade.

## Material and methods

### Ethics statement

All animal work was performed according to European regulations in compliance with FELASA guidelines and regulations. In Greece these consist of the Presidential Decree (160/91) and law (2015/92) and Presidential Decree 56/2013. The experiments were carried out in a certified animal facility license (EL91-BIOexp-02) and the protocol has been approved by the FORTH Ethics Committee and by the Prefecture of Crete (license number # 93491, 30/04/2018). Animal work was approved by the state authorities (Regierungspräsidium Karlsruhe).

### Experimental animals

6–10 week-old Theiler’s Original (OlaTO) of either sex (provided by FORTH in-house certified Animal Breeding Facility) and 6–8 weeks old female NMRI mice (from Janvier Labs) were used for rearing of the parasites and infection of mosquitoes. The procedures are of mild severity and the numbers of animals used are minimized by incorporation of the most economical protocols. Opportunities for reduction, refinement and replacement of animal experiments are constantly monitored and new protocols are implemented whenever possible.

### Bioinformatics analyses

All gene models were from http://www.plasmodb.org/ and http://www.eupathdb.org/. ClustalW and boxshade were performed at http://www.ch.embnet.org/. The phylogenetic tree was reconstructed using the maximum likelihood method implemented in the PhyML program (v3.1/3.0 aLRT following sequence alignement with MUSCLE (v3.8.31) at phylogeny.fr under default settings [12].

### Expression profiling by Reverse Transcriptase PCR

Total RNA was extracted from indicated parasite stages using TRIzol reagent following the manufacturer’s instructions. cDNA was generated with SuperScript II Reverse Transcriptase (RT) in the presence of oligo d(T) and random hexamers; negative controls included omission of RT. Genomic DNA samples were amplified as PCR primer controls. *pbanka_040730 (omd)* was amplified with primers g1086 and g1142; *hsp70* with primers g0258 and g0259; *p28* with primers g0115 and g0116; *trap* with primers g0432 and g0433. Primer sequences are found in S1 Table.

### Generation of GFP-tagged mutant

Plasmids for transfection are based on the CITH::GFP plasmid [13]. Briefly, the *cith* gene part was replaced with the one for PBANKA_040730 with a SwaI-BamHI digested PCR amplicon using primers g1013 and g1061 resulting in plasmid pLIS0171. Prior to transfection the plasmid was linearized with BsmI. Transfectants were selected with pyrimethamine in drinking water. Successful transfectants were cloned by limiting dilution according to established methods [14]. Genotyping was performed by PCR. The presence of the GFP-tagged transgene was shown by Western blot with a monoclonal anti-GFP antibody. Primer sequences are found in S1 Table.

### Generation of *omd(-) cl1*

Plasmids for transfection are based on the *cith* knock out plasmid [13]. pLIS0073 contains as 5’TR the PCR amplicon using primers g0768 and g0769, and as 3’TR the PCR amplicon using primers g0770 and g0771. Prior to transfection pLIS0073 was linearized with KpnI and KspI. Transfection and genotyping was carried out as described above. Primer sequences are found in S1 Table.

### Generation of *omd(-) cl2*

A plasmid designed for *P*. *berghei* gene deletions was modified as follows. The 5’ targeting fragment (813 bp) was amplified with primers 040730-S1 and 040730-S2 and cloned in the restriction sites ApaI-XhoI while the 3’ targeting PCR fragment (807 bp) was amplified with primers 040730-D1 and 040730-D2 and inserted in KpnI-NotI restriction sites. The pKO-040730 plasmid was linearized with ApaI and NotI. Transfection was carried out as above. PCR and Southern blot analyses were performed to verify the successful deletion of the gene. Primer sequences are found in S1 Table.

### Transmission to the mosquito vector

In a standard feeding assay 100 female *Anopheles stephensi* mosquitoes were allowed to feed for 20 minutes on two infected and anaesthetized [Ketamin/Xylazin (2.5mg/0.25mg)] mice 3 days after transfer of 20,000,000 infected RBC into a naïve mouse. Post-infection mosquitoes were kept in an incubator set to 21°C. Oocysts were counted between days 10 and 14 after staining of dissected midguts with 0.1% mercurochrome in PBS or non-stained under the light microscope.

### Microscopy

Live cell imaging of fluorescent parasites was either performed on a Zeiss Axiovert 200 with a magnification of 25x/63x or on a Nikon spinning disc microscope using a 100x objective. Nuclei were visualized with Hoechst.

### *In vitro* ookinete formation and gliding motility

Ookinete conversion experiments were performed by counting ookinetes and round cells after labelling with the antibody against the surface protein Pbs21 as described [13,15]. Imaging of ookinete motility was performed after mixing the ookinete culture (20 hour) 1:1 with Matrigel (BD Bioscience) on glass slides. Ookinetes were imaged using a Zeiss Axiovert 200 microscope.

### Scanning electron microscopy

The material was allowed to adhere to 0.1% poly-L-lysine-coated glass cover slips for 20 minutes at room temperature. The cells were post-fixed at room temperature for 1 h in a solution containing 1% OsO_4_ in 0.1 M cacodylate buffer, pH 7.2. The material was then washed, dehydrated in an ethanol series (15%, 30%, 50%, 70%, 90% and 100%), critical point-dried in CO_2_ and mounted on specimen stubs. Stubs were sputtered with a thin layer of gold and observed in a Zeiss LEO 1530 scanning electron microscope.

### Indirect immunofluorescence assay (IFA)

Ookinetes were fixed in 4% paraformaldehyde for 1 hour, washed in PBS and incubated for 10 min with 0.1% Triton X-100 in PBS. Samples were washed with PBS and blocked in 3%BSA/PBS for 1 h, followed by incubation with the primary antibodies overnight at +4°C. Secondary antibodies were conjugated with Alexa Fluor 488 and Alexa Fluor 568. Controls were included to exclude non-specific binding of the secondary antibodies; they were all negative. Samples were viewed in a Zeiss Axioskop 2 plus microscope. Images were analyzed with ImageJ software (http://rsbweb.nih.gov/ij/).

### Antibodies

Antibodies directed against CTRP [3], SOAP [6], actin I [16], GAP45 [17], enolase [18] and Pbs21 [19] have been described previously. GFP was detected with a monoclonal antibody obtained from Roche while secondary Alexa-conjugated antibodies were from Invitrogen.

## Results

### OMD is conserved in the *Plasmodium* genus and expressed in gametocytes and mosquito stage parasites

**Fig 1:**
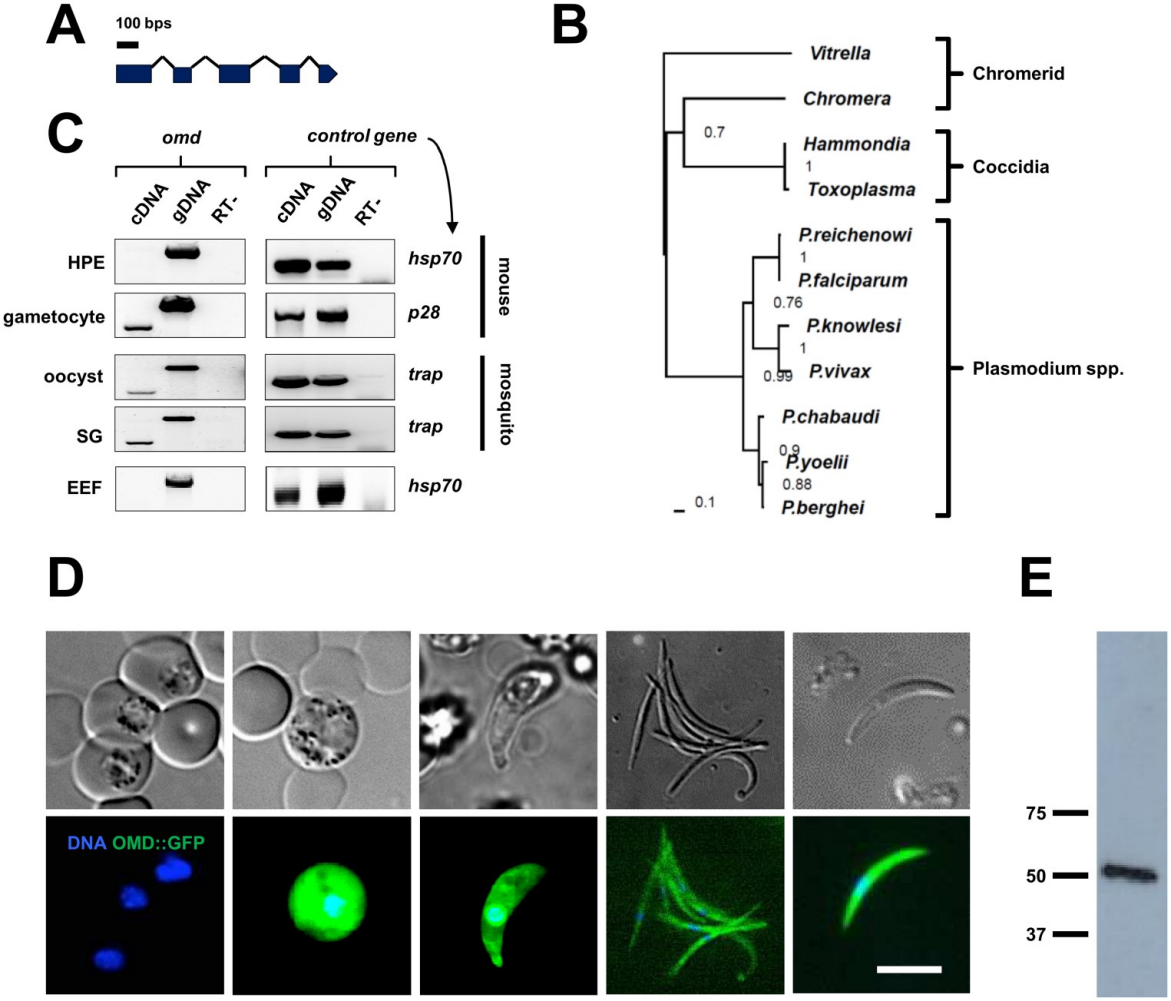
Ookinete motility deficient (OMD) is expressed in gametocytes and mosquito stage parasites. **(A)** Gene model of *Plasmodium berghei* gene PBANKA_040730, here named *ookinete motility deficient (omd)*. **(B)** Phylogenetic tree using the maximum likelihood method calculated from protein sequence alignment of OMD from selected *Plasmodium* species [*P*. *berghei* (PBANKA_0407300), *P*. *falciparum* (PF3D7_0309100), *P*. *reichenowi* (PRCDC_0308400), *P*. *yoelii* (PY17X_0409700), *P*. *chabaudi* (PCHAS_0408200), *P*. *vivax* (PVX_119570), and *P*. *knowlesi* (PKNH_0833600)], *Vitrella brassicaformis* (Vbra_1240), *Chromera velia* (Cvel_12263), *Hammondia hammondi* (HHA_261690) and *Toxoplasma gondii* (TGGT1_261690). Node support values are indicated. **(C)** Transcriptional profiling of *omd* by Reverse Transcriptase-PCR. The gene was found to be expressed in gametocytes, oocysts, and sporozoites but not in liver stage extra-erythrocytic forms (EEF). No signal was seen in mixed blood stages of the strain HPE that does not form gametocytes. Controls with stage-specific transcripts of known genes are shown in right panel and also include samples processed in the absence of reverse transcriptase (RT-) and amplification from genomic DNA. **(D)** Live cell imaging of OMD::GFP in blood and mosquito stage parasites. DNA was stained with Hoechst. Scale bar 5μm. **(E)** Protein size was determined by western blot of OMD::GFP using an α-GFP antibody.

The gene PBANKA_0407300, from here on onwards named *ookinete motility deficient* and abbreviated *omd*, was highlighted in a group of motility and invasion-related genes such as *gap45*, *tlp1*, *celtos* and *spect2*, whose transcription was rapidly downregulated during sporozoite to liver stage development [20]. The gene has five exons (Fig 1A) and encodes a 176 amino acids long protein with a predicted N-terminal signal peptide. The protein is highly conserved within the *Plasmodium* genus (Fig 1B, S1A Fig) suggesting a conserved function in rodent and human malaria parasites. BLASTP searches at eupathdb.org with default settings revealed potential, but highly divergent proteins in *Toxoplasma gondii* and the ancestral, non-parasitic alveolates *Chromera velia* and *Vitrella brassicaformis* (S1B and S1C Fig). In addition, a protein structure homology-modeling approach [21] identified structural similarities with the chaperone Mesd from mouse (https://www.uniprot.org/uniprot/Q9ERE7), a homolog of the *Drosophila melanogaster Boca* protein (S1D Fig).

At the transcriptome level, RNA-seq had identified strong gametocyte expression of *omd* in *P*. *berghei* with little evidence for transcription in asexual stage parasites or ookinetes [22]. To address independently the transcriptional profile of *omd* throughout the entire life cycle, we performed Reverse Transcriptase-PCR analyses on stage-specific cDNAs and found *omd* transcribed in gametocytes and all mosquito stages. No expression was detected in mixed blood stages of the HPE strain that does not form gametocytes, nor in exoerythrocytic liver stage forms (EEF) (Fig 1C) suggesting a role for the protein restricted to transmission stages. The orthologous protein *P*. *falciparum* PF3D7_0309100 was detected in stage I/II (3D7) and stage V (NF54) gametocytes while there is no proteome evidence for expression in asexual stage parasites in either species [22]. To examine the expression profile of OMD and determine its subcellular localization we introduced a C-terminal GFP-tag into the endogenous locus by standard plasmid transfection methodologies [14], thus keeping the fusion protein under the control of the native promoter (S2 Fig). Consistent with the RT-PCR data, fluorescence was apparent in gametocytes, ookinetes and oocysts, as well as in midgut and salivary gland sporozoites, but never detected in asexuals of the *omd*::*gfp* line (Fig 1D). The OMD::GFP signal appeared mostly uniform and cytoplasmic. Western blot analysis showed that the fusion protein had the expected molecular weight of 48 kDa (Fig 1E). We verified that *omd*::*gfp* parasites transmitted readily into the mosquito vector, showing that the tag did not interfere with the normal function of the protein; the *omd*::*gfp* parasite line produced an average of 6500 salivary gland sporozoites (n = 20) compared to 9450 (n = 25) in the WT control infection.

### OMD is dispensable for asexual blood stages and ookinete formation but essential for gliding motility and mosquito infection

**Fig 2:**
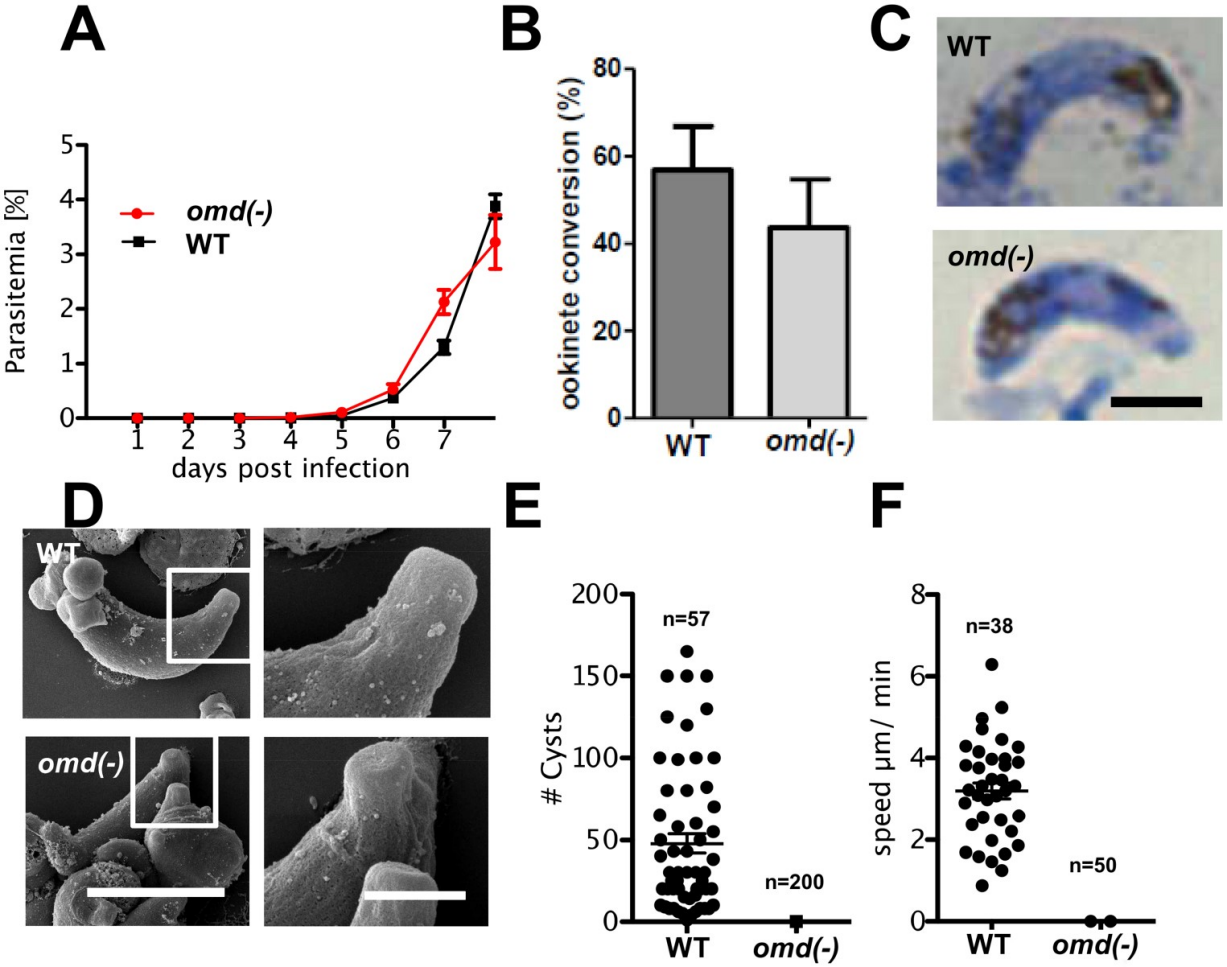
OMD depleted ookinetes lack gliding motility. **(A)** Growth curves of WT and *omd(-)* asexual stage parasites. Average values of four replicates for each strain are shown. Error bars denote s.e.m. **(B)** Ookinete conversion measured as the percentage of Pbs21-immunolabeled female gametes and zygotes that develop into ookinetes. The data are the average of four independent experiments. Error bar denotes s.e.m. revealing no statistical significant difference by Student’s t-test. **(C)** Giemsa-stained images of WT and mutant ookinete types. Scale bar = 5 μm. **(D)** Scanning Electron Microscopy (SEM) imaging of WT and mutant ookinetes, with detail of apical part. Scale bars, left panel 5 μm, right panel 1 μm. **(E)** Oocyst numbers of WT (n = 57) and mutant (n = 200) counted 12 days after standard mosquito feeding assay. Pooled data from two experiments. Error bars denote s.e.m. **(F)** Gliding motility speeds of WT (n = 38) and mutant ookinetes (n = 50) in Matrigel. Error bars denote s.e.m. *** P<0.0001, Student’s t-test. See also Supporting information S1 and S2 Movies.

To address the role of OMD during malaria transmission we generated two independent *omd(-)* knock out clones *omd(-)cl1* and *omd(-)cl2* (S3 and S4 Figs). Null mutants displayed normal asexual blood stage development (Fig 2A) consistent with genome-wide phenotype data [23]. Ookinete formation (measured as the percentage of female gametes that developed into ookinetes) did not reveal a significant difference between mutant and WT parasite development (Fig 2B) with ookinete morphologies similar as revealed by Giemsa-stained smears (Fig 2C) and scanning electron microscopy analysis (Fig 2D). Ookinetes were also formed *in vivo* as revealed by Giemsa stained smears of mosquito midguts formed 24 h after feeding on an infected mouse (S5 Fig). *In vivo* transmission experiments, where mosquitoes are allowed to blood feed on mice infected with the *omd(-)cl1* mutant, did not result in the establishment of oocysts in three independent feedings; WT parasites on the other hand transmitted normally (Fig 2E). Data for *omd(-)cl2* were consistent with these results (WT 105 oocysts in 15 mosquitoes, *omd(-)cl2* 0 oocysts in 60 mosquitoes, pooled data from two feedings). As a consequence, *omd(-)* sporozoites were never detected and no transmission to naïve mice was found in three independent experiments of *omd(-)cl2*. The WT controls in each case were transmitted normally and identified in Giemsa stained blood smears after five days.

The failure to establish oocysts entirely or in reduced numbers has previously been observed in parasites having defects in motility [3,9,24]. In order to investigate whether this was the case for *omd(-)* mutant ookinetes we performed gliding motility assays. Comparing motility of WT and *omd(-)cl1* ookinetes by live microscopy showed that WT parasites moved at a velocity of 1–7 μm.min^-1^ while mature *omd(-)* ookinetes were completely devoid of productive motility (Fig 2F); at most they displayed stretching of the ookinete. Identical results were obtained from *omd(-)cl2* (S1 and S2 Movies). These results indicate a key role for OMD in motility and its absolute requirement for mosquito infection.

### Key motility and invasion factors are not affected in the *omd(-)* mutant

**Fig 3:**
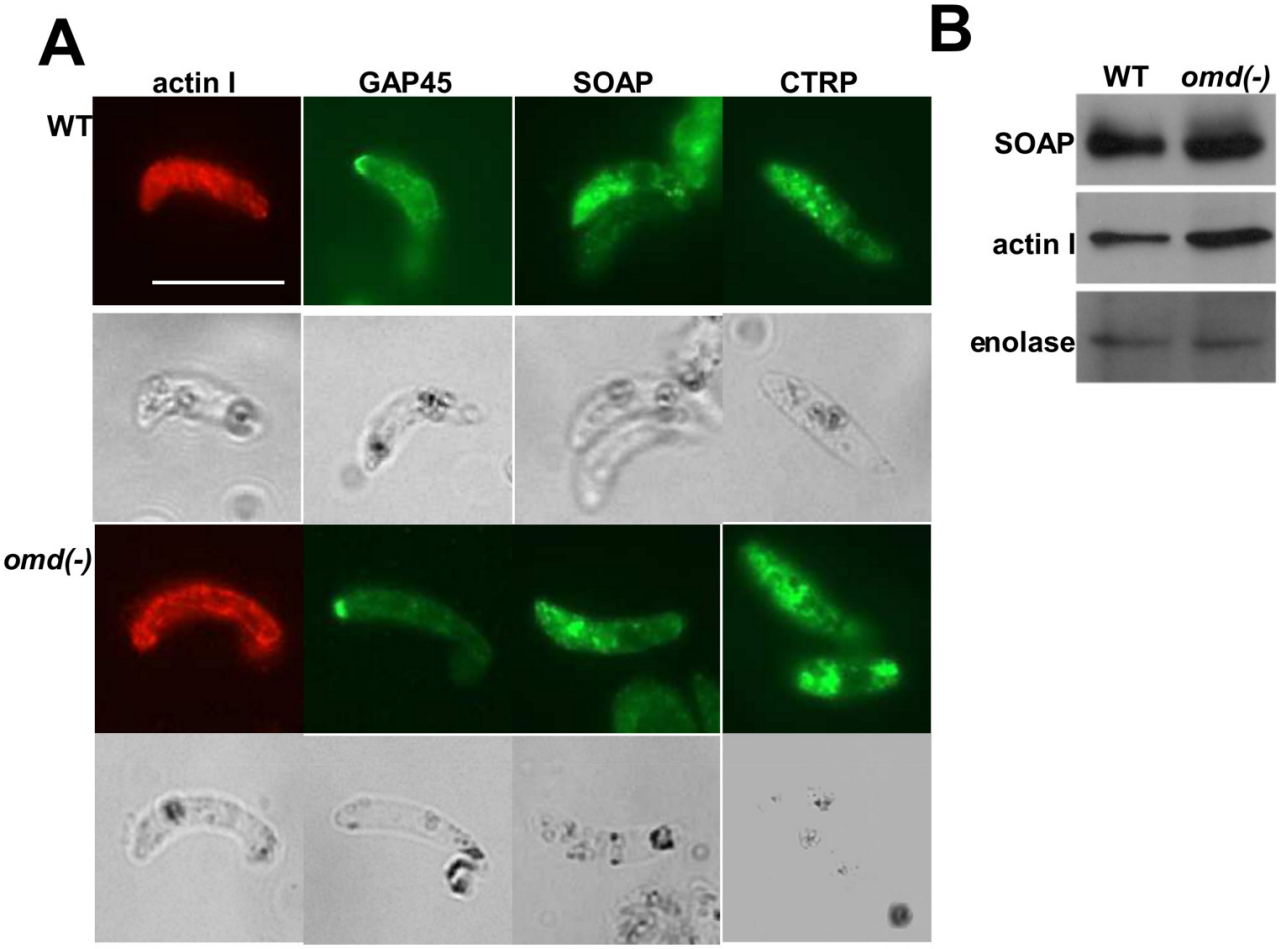
Key invasion factors and motility factors are normally expressed in *omd(-)* mutant ookinetes. **(A)** IFA of WT (top) and *omd(-)* ookinetes using antibodies directed against the glideosome components actin I and GAP45 as well as against the micronemal proteins CTRP and SOAP. In all pictures the apical end is towards the left. Scale is the same in all pictures, scalebar in A 10 μm. **(B)** Western blot of crude extracts of WT and *omd(-)* ookinetes. The blot was probed with antibodies against SOAP (top), actin I (middle) and enolase as a loading control (bottom).

We next attempted to investigate the defect in ookinete motility of the *omd(-)* mutant and the failure to transmit to the mosquito vector by examining the subcellular localization of key components involved in ookinete motility for which antibodies were available. We explored the subcellular distribution of actin I, an essential protein for ookinete motility, using an antibody that we have previously reported as detecting filamentous F-actin in ookinetes [16]. There was no obvious difference in the pattern of actin I in the mutant compared to the WT (Fig 3A) nor in the amount of this protein, determined by Western blot, in the mutant compared to WT (Fig 3B). Labeling of GAP45, a key constituent of the inner membrane complex (IMC) and anchor of the gliding motility apparatus, primarily detected in the apical tip of ookinetes, revealed that this protein was also localized normally in *omd(-)* parasites (Fig 3A). SOAP and the adhesion CTRP were tested as markers for the formation of micronemes [3,6]. In *omd(-)* ookinetes CTRP and SOAP (Fig 3A) were detected primarily in the apical end of the ookinete similar to the WT. Furthermore, Western blot analysis of SOAP did not reveal any significant differences in the amount of SOAP protein (Fig 3B). Taken together this suggests that the failure of motility in the *omd(-)* mutant is not due to gross mis-localization of either micronemal proteins or components of the motility machinery. The lack of antibodies against other proteins involved in motility did not allow us to investigate this further.

## Discussion

The data presented here show that OMD is expressed in gametocytes and all parasite forms of the mosquito, and plays an essential role for ookinete biology. In the absence of the protein ookinetes are formed that completely lack productive motility although some stretching movements similar to those of mutants lacking the micronemal protein CTRP and CDPK3 [3,25] were detected. Consequently, the mutant parasites failed to establish oocysts after transmission to the mosquito. Our attempts to detect mis-localization of micronemal proteins or components of the motility machinery did not reveal any striking differences comparing the mutant to the WT. However, as we only looked at a limited number of proteins for which antibodies are available, we cannot exclude that other proteins are affected in the absence of OMD.

One possible clue to the function of OMD is a C-terminal KDEL endoplasmatic reticulum (ER) retrieval motif, which it shares with the chaperones endoplasmin/GRP94, HSP70/GRP78 and protein disulfide isomerase [26,27]. This tetrapeptide motif mediates trafficking of these proteins from the Golgi complex back to the ER after recognition by KDEL receptors. Apart from this targeting signal, OMD is devoid of defining domains that could indicate a possible function. In the genus *Plasmodium* the protein is highly conserved, but related proteins may exist in other apicomplexans such as *T*. *gondii* and *H*. *hammondi*, and the related phototrophic ancient alveolates *Chromera velia* and *Vitrella brassicaformis*. A homology modeling approach revealed weak similarities with the ER-resident LDLR (low-density lipoprotein receptor) chaperone MESD which is required for the correct folding of beta-propeller and EGF domains [28]. Based on this weak sequence similarity, but without any experimental support at this time, we hypothesize that OMD may function as a life cycle stage-specific chaperone for a yet to be identified crucial gliding motility factor in the ookinete; to date our attempts to pinpoint such a function has been unsuccessful. Another possibility is involvement in the signaling events required for motility [8–10]. Future work will be required to dissect the role of this intriguing protein.

## Supporting information

S1 Fig Multiple sequence alignments of selected OMD homologs. **(A)** PF3D7 *P*. *falciparum*, PRCDC *P*. *reichenowi*, PBANKA *P*. *berghei*, PY17X *P*. *yoelii*, PCHAS *P*. *chabaudi*, PVX *P*. *vivax* and PKNH *P*. *knowlesi*. **(B)** TGGT1 *Toxoplasma gondii*, HHA *Hammondia hammondi*, Vbra *Vitrella brassicaformis*, Cvel *Chromea velia* and *P*. *berghei* PBANKA_040730 (OMD) alignment. **(C)** *Toxoplasma gondii* and *P*. *berghei* alignment. **(D)** *P*. *berghei* OMD, mouse MESD and *Drosophila melanogaster* Boca alignment.
(PDF)

S2 Fig C-terminal GFP-tagging of *Plasmodium berghei* PBANKA_040730 in the *omd*::*gfp* line. **(A)** Schematic representation of wildtype (top), transfection plasmid and mutated *omd* (bottom) loci. The plasmid construct was digested with the restriction enzyme BsmI to allow integration. The plasmid contains a GFP encoding sequence fused in frame to the *omd* targeting region and the TgDHFR/TS antifolate pyrimethamine resistance cassette Primer pairs and expected amplicon sizes are indicated. Positions of primers used in PCR genotyping are shown. **(B)** PCR genotyping of *omd*::*gfp* indicating the primer pairs and amplicon sizes. Wildtype and *omd*::*gfp* genomic DNA were used as templates.
(PDF)

S3 Fig Gene deletion and genotyping of *omd* (PBANKA_040730) knock-out mutant *omd(-)cl1*. **(A)** Schematic of wildtype (top), transfection plasmid and mutant (bottom) loci. Top: The position of 5’ and 3’ flanking regions (TR) (orange) are indicated as well as the TgDHFR/TS antifolate cassette (5’ and 3’ flanking regions in blue, ORF red). The plasmid was digested with restriction enzymes KpnI and SacII. The positions of all primers used in generating plasmids and for genotyping are indicated. **(B)** PCR genotyping of *omd(-)cl1* indicating the primer pairs and amplicon sizes. Wildtype and *omd(-)cl1* genomic DNA were used as templates.
(PDF)

S4 Fig Gene deletion and genotyping of *omd* (PBANKA_040730) knock-out mutant *omd(-)cl2*. **A** Schematic of wildtype (top), transfection plasmid and mutant (bottom) loci. Top: The position of 5’ and 3’ flanking regions (TR) (orange) are indicated as well as the TgDHFR/TS antifolate cassette (5’ and 3’ flanking regions in blue, ORF red). The plasmid was digested with restriction enzymes ApaI and NotI. The positions of all primers used in generating plasmids and for genotyping are indicated. **B** PCR genotyping of *omd(-)cl2* indicating the primer pairs and amplicon sizes. Wildtype (WT) and *omd(-)cl2* genomic DNA were used as templates. **C** Southern blot confirmed the correct integration. gDNA of two different populations, after transfection and before cloning (lanes 1,2) and WT (lane 3), were analyzed. The probe corresponds to the 5’ target region. The expected fragments of the two mixed populations are indicated in grey in A. As expected only the 4776 bp band was detected in the WT. The sample in lane 2 was used for the cloning of *omd(-)cl2* line.
(PDF)

S5 Fig *Omd(-)* ookinetes formed *in vivo*. Mosquitoes were fed to mice infected with *omd(-)cl2*. 24 h after feeding midguts were dissected and smeared on a glass slide followed by staining with Giemsa. Eight representative ookinetes are shown. Scale bar, 5 μm.
(PDF)

S1 Table Oligonucleotide primers used in the current study. (DOCX)

S1 File The ARRIVE guidelines checklist. (PDF)

S2 File Raw data for graphs in Fig 2. (XLSX)

S1 Movie WT ookinete imaged moving in matrigel. (AVI)

S2 Movie *Omd(-) cl2* imaged in matrigel. No movement over a distance was detected, though stretching was observed.
(AVI)
