# Supplementary material for: Malaria transmission through the mosquito requires the function of the OMD protein
Source: PLoS One. 2019 Sep 25;14(9):e0222226. doi: 10.1371/journal.pone.0222226 (PMC6760768; doi:10.1371/journal.pone.0222226)
Supplement: S1 Fig — (A) PF3D7 P. falciparum, PRCDC P. reichenowi, PBANKA P. berghei, PY17X P. yoelii, PCHAS P. chabaudi, PVX P. vivax and PKNH P. knowlesi. (B) TGGT1 Toxoplasma gondii, HHA Hammondia hammondi, Vbra Vitrella brassicaformis, Cvel Chromea velia and P. berghei PBANKA_040730 (OMD) alignment. (C) Toxoplasma gondii and P. berghei alignment. (D) P. berghei OMD, mouse MESD and Drosophila melanogaster Boca alignment. (PDF) [file pone.0222226.s001.pdf]

A

```
PF3D7_0309100     1 MNMIMKIYYIFLLFVFILIERCVDND----VINKTQENYLDHRDFEKDMDELDLSGLEYD
PRCDC_0308400     1 MKMIMKIYYIFLLFVFILIERCVDND----VINKTQENYLDHRDFEKDMDELDLSGLEYD
PBANKA_0407300    1 ----MKIYYLFFLLILIQLN-LVNNQ----DLNQPTNDNPLNRDFEKDMEELDLSGLEDD
PY17X_0409700     1 ----MRIYYLFFFLILIQLN-LVNNQ----NLNQPNNANPLNRDFEKDMEELDLSGLEDD
PCHAS_0408200     1 ----MKIYYLFFLLILIQLN-LVNSQ----DLNQPKNDNPLNRDFEKDMEELDLSGLEDD
PVX_119570        1 ----MKPLHLLVLLLPLGSSGGGDS----------GGVVLPRDFEKDMSELDLSGLEGD
PKNH_0833600      1 ----MKPFHLLVLLIFPLARFGRGESPDGGKGDGSNGGVVFPRDFDKDMNELDLSGLEED

PF3D7_0309100    57 IENVEEIAQKDSYNYVTQIYVYIRIDEEYKKYINHLELMRLGQRFMTILQNAMINVQLKK
PRCDC_0308400    57 IENVEEIAQKDSYNYVTQIYVYIRIDEEYKKYINHLELMRLGQRFMTILQNAMINVQLKK
PBANKA_0407300   52 IENIEEIAYKDSYTYVTQIYIYVRIDGDYSIYKSHSNLMKLGEKYMTLLQNAMLNVQLKK
PY17X_0409700    52 IENIEEIAYKDSYTYLTQIYIYVRIDGDYSMYKSHSNLMKLGEKYMTLLQNAMLNVQLKK
PCHAS_0408200    52 IENIDEIAYEDNYTYVTHIYIYVRIDGDYSIYKNHSNLMKLGEKYMTLLQNAMMNVQLKK
PVX_119570       47 IEGIDEVAHRDYYQYVTQLYVYVRVDGKYRTFRNHPQLIRLGEKYMTLLQNAMINVQLRR
PKNH_0833600     57 IEGIEEIAHTDYYQYVTQLYVYVRIDKKYSTFRNHLQLIRLGEKFMTLLQNAMINVQMKK

PF3D7_0309100   117 TGTGIFTCFYENKSITDDLATYFLIQKEVDFIEVGFDKRYPEGRHAPITDSDMRVFIK--
PRCDC_0308400   117 TGTGIFTCFYENKSITDDLATYFLIQKEVDFIEVGFDKRYPEGRHAPITDSDMRVFIK--
PBANKA_0407300  112 NGIGEFICYYQKKDITENLATYFLIQKEIDYIQIGFEKRFPEGRISPIVDAEKRKTSSEN
PY17X_0409700   112 NGVGEFICYYQKKDITENLATYFLIQKEIDYIQIGFEKRFPEGRISPIVDAEKRKTSSEN
PCHAS_0408200   112 NGVGEFICYYQKKEITENLATYFLIQKEIDYIQIGFEKRFPEGRTFPIVDADKRKTTSEN
PVX_119570      107 TGIGIFTCFYQDKAITENLVTYFLMQKEVDFLEVGFDRRFPEGRSAPIVDVDGRVTPQGH
PKNH_0833600    117 TGVGIFTCFYQDKAITENLVTYFLMQKEVDFLEVGFDRRFPEGRSAPFVDVDERIDPQEK

PF3D7_0309100   177 -NEEL
PRCDC_0308400   177 -NEEL
PBANKA_0407300  172 IKDEL
PY17X_0409700   172 IKDEL
PCHAS_0408200   172 VKDEL
PVX_119570      167 PHEEL
PKNH_0833600    177 TDEEL
```

B

```
TGGT1_261690      1 MDRMTREGRQAEQAWVVLALLFHVLNVLSNGTAGRLQYFCARAEEYQPPRKHWKEFIIED
HHA_261690        1 MDRMTREGRQAGEAWVVLALLFLVLNVLSNGTAGGPQHVCARAEEYQPPRKHWKEFVIED
Vbra_1240         1 MASVSLYVRCSLLLALVLLLVFPIRESAAARED-------------------------DD
Cvel_12263        1 MSMVMVPLPSALLQACLAFQVTWCVTASPSGSYPG----------------IDETFVIED
PBANKA_0407300    1 --MKIYYLFFLLILIQLNLVNNQDLNQPTNDNPLN-----------------------RD

TGGT1_261690     61 AAKDIEELDVGDINDDQIDLPPDFPR------------SASPADSFRIDMDDEGDWAQTM
HHA_261690       61 AAKDIEELDVGDINDDQIDLPPDFPR------------SASPADSFRIDMDDEGDWAQTM
Vbra_1240        36 DEDIDEEEDDDDVRAARRRREAAKAR------------MEQMQQMPKFDPKNFNPQAIFD
Cvel_12263       45 VEKDRAELDVGDLTDWTVDTPPNYKPKRKDLQGESERGRKREKKKRKQREGSQVEESVVD
PBANKA_0407300   36 FEKDMEELDLSGLEDDIEN----------------------------------IEEIAYK

TGGT1_261690    109 GTHFGEHLLIVNLRLDGE-FELYGDPDFTGAVLSKWVAMLKNAKADVRVYSVESARAIIN
HHA_261690      109 GTHFGEHLLIVNLRLDGE-FELYGDPDFTGAVLSKWVAMLKNAKADVRVYSVESARAIIN
Vbra_1240        84 AQRNKPQMTFATLKFEYE-EKEKQE---TEKIAVRWRSMLEMGHIQVQAYAVDPGKILLM
Cvel_12263      105 SAKKGVATTLVFLKWSFCGQKGKRTAKDLAELGRVWLDVLGSIGVGVQLHILDTCQLAAI
PBANKA_0407300   62 DSYTYVTQIYIYVRIDGD-YSIYKSHSNLMKLGEKYMTLLQNAMLNVQLKKNGIGEFICY

TGGT1_261690    168 LRNTADIGIVRRFLAQQPDVDYWTIDGNRFFPPGRSEPYLTEGER---------------
HHA_261690      168 LRNTADIGIVRRFLAQQPDVDYWTIDGNRFFPPGRSEPYLTEGER---------------
Vbra_1240       140 TQNADQADKVREFALAQRETDWFEINRKRFFPDDRDGPLVDYDVR---------------
Cvel_12263      165 TRREPDKEVLWQFLLAKDEVDFLEFGTETIFPYGRTKPITSPAERKTLMTQLEKFEDSRG
PBANKA_0407300  121 YQKKDITENLATYFLIQKEIDYIQIGFEKRFPEGRISPIVDAEKR---------------

TGGT1_261690    213 -------AKRIRAMYEGDPLFDNKKKDEL
HHA_261690      213 -------AKRIRAMYEGDPLFDKKKKDEL
Vbra_1240       185 -------RKARDARKAK-----KEGKTEL
Cvel_12263      225 FREQREQQQQRTQATKEAARKKRTEEL
PBANKA_0407300  166 ------------------KTSSENIKDEL
```

C

```
PBANKA_0407300    1 ----------MKIYYLFFLLILIQLNLVNN------------QDLNQPTN---DNPLNRD
TGGT1_261690      1 MDRMTREGRQAEQAWVVLALLFHVLNVLSNGTAGRLQYFCARAEEYQPPRKHWKEFIIED

PBANKA_0407300   36 FEKDMEELDLSGLEDD-IENIEEIAYKDSYTYVTQIYIY---------------------
TGGT1_261690     61 AAKDIEELDVGDINDDQIDLPPDFPRSASPADSFRIDMDDEGDWAQTMGTHFGEHLLIVN

PBANKA_0407300   74 VRIDGDYSIYKSHSNLMKLGEKYMTLLQNAMLNVQLKKNGIGEFICYYQKKDITENLATY
TGGT1_261690    121 LRLDGEFELYGDPDFTGAVLSKWVAMLKNAKADVRVYSVESARAIINLRNTADIGIVRRF

PBANKA_0407300  134 FLIQKEIDYIQIGFEKRFPEGRISPIVDAEKRKTSS-----------ENIKDEL
TGGT1_261690    181 LAQQPDVDYWTIDGNRFFPPGRSEPYLTEGERAKRIRAMYEGDPLFDNKKKDEL
```

D

```
MESD    1 MAASRWLRAVLLFLCASDLLLPPPNAYAADTPGEATPPPRKKKDIRDYNDADMARLLEQ
Boca    1 ------------MQTRLVLLLLALTPLVLAKKFKEEEKPAWAKKDIRDYSEADLERLLDQ
OMD     1 -------------MKIYYLFFLLILIQLNLVNNQDLNQPTNDNPLNRDFEKDMEELDLSG

MESD   61 WEKDDD-IEEGDLPEHKRPSAPIDFSKLDPGKPESILKMIKKGKTLMMFVTVSGNPTEKE
Boca   49 WEEDEEPLEDDELPEHLRPQPKIDLSNLDSKSPEDLLKVSKKGRTLMTFVSVTGNPTREE
OMD    48 LEDDIENIEEIAYKDSYTYVTQIYIYVRIDGDYSIYKSHSNLMKLGEKYMTLLQNAMLNV

MESD  120 TEEITSLWQGSLFNANYDVQRFIVGSDRAIFMLRDGSYAWEIKDFLVSQDRCAEVTLEGQ
Boca  109 SDTITKLWQTSLWNNHIQAERYMVDDNRAIFLFKDGTQAWDAKDFLIEQERCKGVTIENK
OMD   108 QLKKNGIGEFICY---YQKKDITENLATYFLIQKEIDYIQIGFEKRFPEGRISPIVDAEK

MESD  180 MYPGKGGGSKEKNKTKPEKAKKKEGDPKPRASKEDNRAGSRREDL
Boca  169 EYPGVNAK-----------------------------------KEDL
OMD   165 RKTSSENI-----------------------------------KEDL
```

S1Fig
