# Supplementary figures and images for: Malaria transmission through the mosquito requires the function of the OMD protein
Source: PLoS One. 2019 Sep 25;14(9):e0222226. doi: 10.1371/journal.pone.0222226 (PMC6760768; doi:10.1371/journal.pone.0222226)

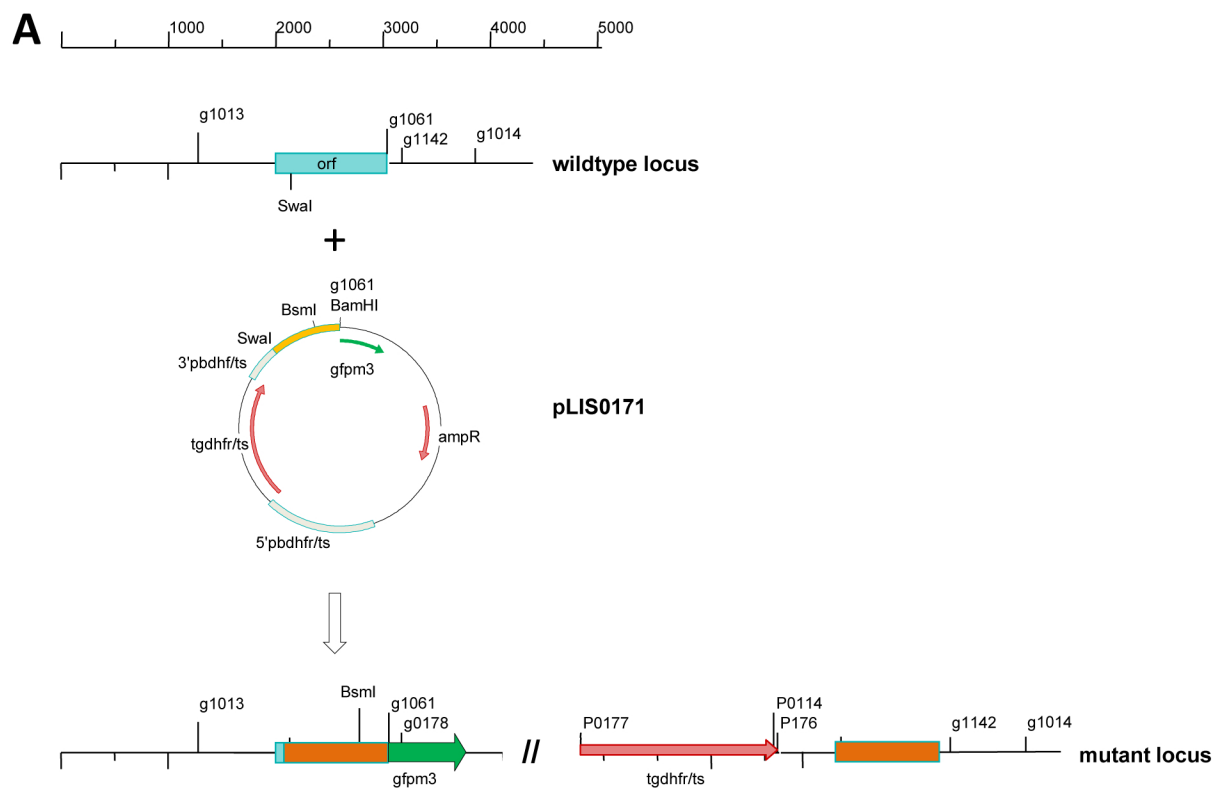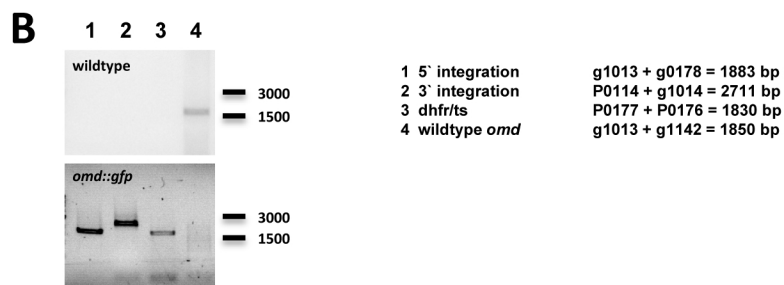

**S2Fig.**

Supplement: S2 Fig — (A) Schematic representation of wildtype (top), transfection plasmid and mutated omd (bottom) loci. The plasmid construct was digested with the restriction enzyme BsmI to allow integration. The plasmid contains a GFP encoding sequence fused in frame to the omd targeting region and the TgDHFR/TS antifolate pyrimethamine resistance cassette Primer pairs and expected amplicon sizes are indicated. Positions of primers used in PCR genotyping are shown. (B) PCR genotyping of omd::gfp indicating the primer pairs and amplicon sizes. Wildtype and omd::gfp genomic DNA were used as templates. (PDF) [file pone.0222226.s002.pdf]

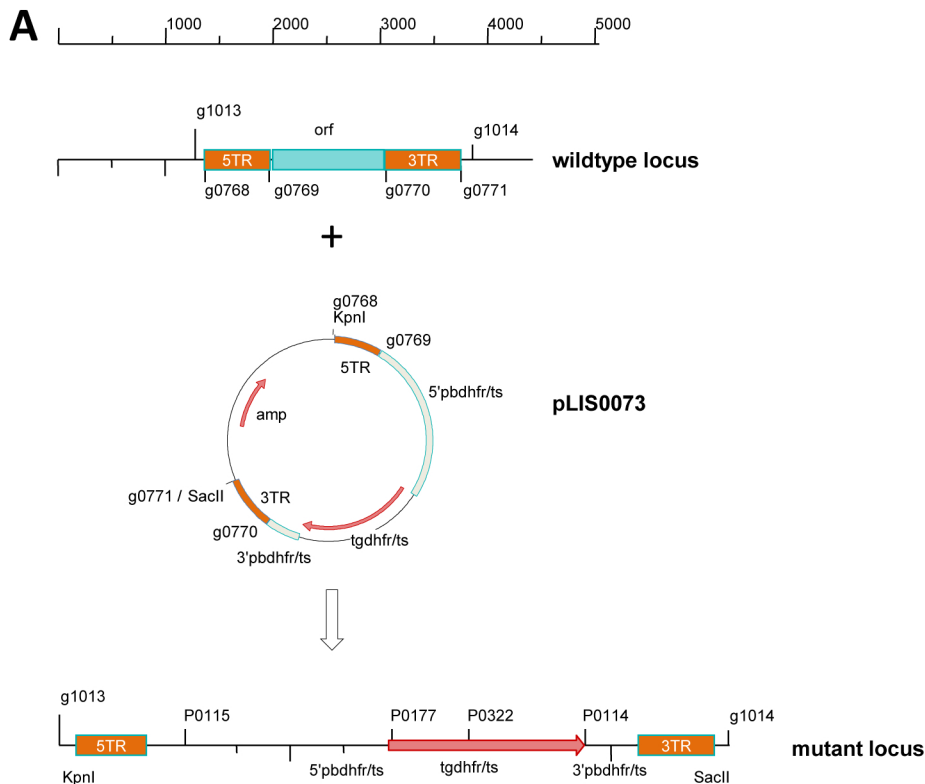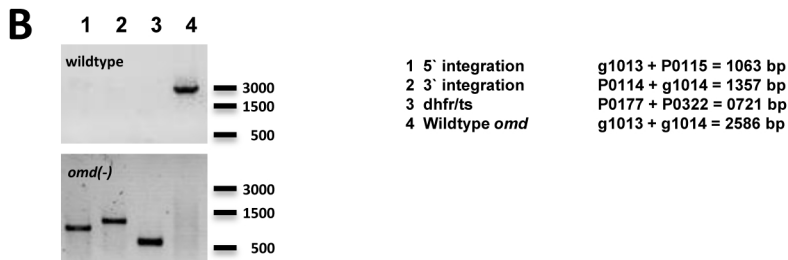

**S3Fig.**

Supplement: S3 Fig — (A) Schematic of wildtype (top), transfection plasmid and mutant (bottom) loci. Top: The position of 5’ and 3’ flanking regions (TR) (orange) are indicated as well as the TgDHFR/TS antifolate cassette (5’ and 3’ flanking regions in blue, ORF red). The plasmid was digested with restriction enzymes KpnI and SacII. The positions of all primers used in generating plasmids and for genotyping are indicated. (B) PCR genotyping of omd(-)cl1 indicating the primer pairs and amplicon sizes. Wildtype and omd(-)cl1 genomic DNA were used as templates. (PDF) [file pone.0222226.s003.pdf]

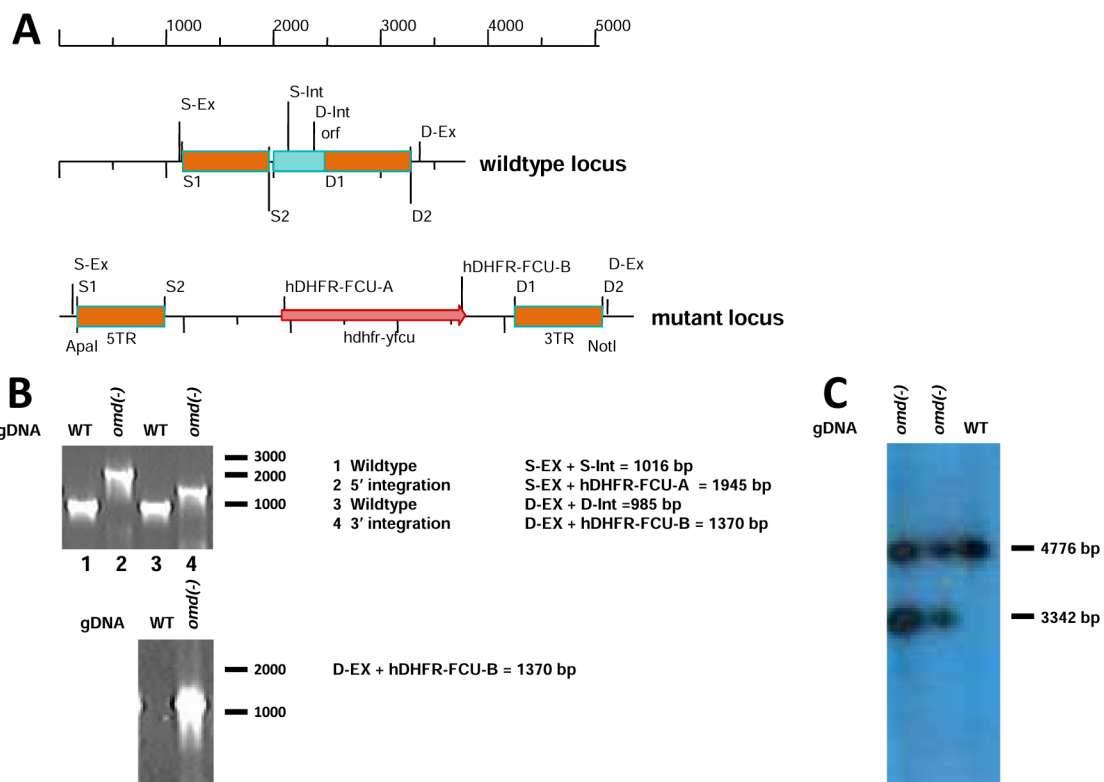

**S4Fig.**

Supplement: S4 Fig — A Schematic of wildtype (top), transfection plasmid and mutant (bottom) loci. Top: The position of 5’ and 3’ flanking regions (TR) (orange) are indicated as well as the TgDHFR/TS antifolate cassette (5’ and 3’ flanking regions in blue, ORF red). The plasmid was digested with restriction enzymes ApaI and NotI. The positions of all primers used in generating plasmids and for genotyping are indicated. B PCR genotyping of omd(-)cl2 indicating the primer pairs and amplicon sizes. Wildtype (WT) and omd(-)cl2 genomic DNA were used as templates. C Southern blot confirmed the correct integration. gDNA of two different populations, after transfection and before cloning (lanes 1,2) and WT (lane 3), were analyzed. The probe corresponds to the 5’ target region. The expected fragments of the two mixed populations are indicated in grey in A. As expected only the 4776 bp band was detected in the WT. The sample in lane 2 was used for the cloning of omd(-)cl2 line. (PDF) [file pone.0222226.s004.pdf]

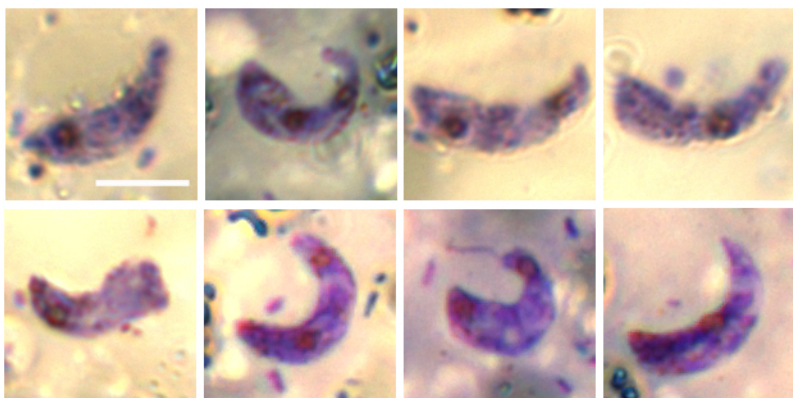

**S5Fig.**

Supplement: S5 Fig — Mosquitoes were fed to mice infected with omd(-)cl2. 24 h after feeding midguts were dissected and smeared on a glass slide followed by staining with Giemsa. Eight representative ookinetes are shown. Scale bar, 5 μm. (PDF) [file pone.0222226.s005.pdf]
