# Supplementary material for: Malaria transmission through the mosquito requires the function of the OMD protein
Source: PLoS One. 2019 Sep 25;14(9):e0222226. doi: 10.1371/journal.pone.0222226 (PMC6760768; doi:10.1371/journal.pone.0222226)
Supplement: S1 Table — (DOCX) [file pone.0222226.s006.docx]

**S1 Table. Primers used for the RT-PCR detection of *omd* transcript and construction and genotyping of the *omd(-)*  and *omd::gfp* mutants**

| Primer name | Primer sequence | Target gene | reference |
| --- | --- | --- | --- |
| **RT-PCR analysis of *omd*** | | |  |
| g1086 | GGAAGAATTGGATTTGTCCG | *omd ORF* | This study |
| g1142 | CTGCAAATTTATATTAGC | *omd 3’ region* | This study |
| g0258 | AAAAGCAAAGCCAAACTTACC | *hsp70 ORF* | This study |
| g0259 | GGATGGGGTTGTTCTATTACC | *hsp70 ORF* | This study |
| g0115 | ATGAATTTTAAATACAG | *p28 ORF* | This study |
| g0116 | GCATTACTATCACGTAAATAAC | *p28 ORF* | This study |
| g0432 | AACATTCACTCCATTCTTCC | *trap ORF* | This study |
| g0433 | CATGTTATTCCAATGCTCAC | *trap ORF* | This study |
| ***Generation of C-terminally GFP-tagged mutant omd::gfp*** | | |  |
| g1013 | AAAGAATTCAGCGAAACTAAATTAGAG | *omd 5’ region* | This study |
| g1061 | AAAGGATCCCAATTCATCTTTTATATTTTC | *omd omd ORF* | This study |
| ***Genotyping of omd::gfp*** | | | |
| g0178 | CCGTATGTTGCATCACCTTCACCC | *gfp* | This study |
| P114 | CCCGCACGGACGAATCCAGATGG | *dhfr/ts* | This study |
| g1014 | TCGACTTAATGTATGTCC | *omd 3’ region* | This study |
| P177 | ATGCATAAACCGGTGTGTCTGG | *dhfr/ts* | This study |
| P176 | CTAGACAGCCATCTCCATCTGG | *dhfr/ts* | This study |
| g1142 | CTGCAAATTTATATTAGC | *omd 3’region* | This study |
| g1086 | GGAAGAATTGGATTTGTCCG | *omd ORF* | This study |
| ***Generation of N-terminally GFP-tagged expression plasmid*** | | | |
| g3193 | AAAGAATTCAAATTATTTTGTGGGC | *omd* 5’ region | This study |
| g3191 | AAAGGATCCAAGCTTATTTAAAGGATTATCATTG | *omd* ORF | This study |
| g3340 | AAAAAGCTTCAAGATTTAAATCAACC | *omd* ORF | This study |
| g3044 | AAAGGCGCCGGCGGCCGCTTGATATTTTAAGAGAAGAG | *omd* 3’ region | This study |
| ***Generation of omd(-)cl1*** | | | |
| g0768 | AAAGGTACCAAATTATTTTGTGGGC | *omd* 5’ TR | This study |
| g0769 | AAAAAGCTTCATTTTATATTAAAAG | *omd* 5’ TR | This study |
| g0770 | AAAGAATTCATATGGAATTTTTTTTAT | *omd* 3’ TR | This study |
| g0771 | AAAGCGGCCGCAGGAATGGATAATGACAG | *omd* 3’ TR | This study |
| ***Genotyping of omd(-)cl1*** | | | |
| g1013 | AAAGAATTCAGCGAAACTAAATTAGAG | *omd 5’ region* | This study |
| P115 | CGCATTATATGAGTTCATTTTACACAATCC | *dhfr/ts* | This study |
| P114 | CCCGCACGGACGAATCCAGATGG | *dhfr/ts* | This study |
| g1014 | TCGACTTAATGTATGTCC | *omd 3’region* | This study |
| P177 | ATGCATAAACCGGTGTGTCTGG | *dhfr/ts* | This study |
| P322 | CCCCGTTGTCTGAGAAGG | *dhfr/ts* | This study |
| ***Generation of omd(-)cl2*** | | |  |
| 040730-S1 | gaccgaattcgggcccGTGTTTCCATTTATGTTATTG | *omd* 5’ TR | This study |
| 040730-S2 | gaccctcgagATGTACATATATGTGTAAGGGG | *omd* 5’ TR | This study |
| 040730-D1 | gaccggtacccccgggGCCACATTTACAAACCCTC | *omd* 3’ TR | This study |
| 040730-D2 | gaccgcggccgcAACCCCGAAATAACACATTTG | *omd* 3’ TR | This study |
| ***Genotyping of omd(-)cl2*** | | | |
| 040730-S-Ex | CGAAATGAAATATGTAATTGTC | *omd 5’ region* | This study |
| 040730-S-Int | gacaaatccaattcttccatg | *omd ORF* | This study |
| hDHFR-FCU-A | cgatgcagtttagcgaacca | *hdhfr-fcu* | This study |
| 040730-D-Ex | caatgactctaatattgatcg | *omd3’ region* | This study |
| 040730-D-Int | GGCTGTCTTTATGTCCATTTGG | *omd ORF* | This study |
| hDHFR-FCU-B | GTTGGGTGACTTTGGTGACA | *hdhfr-fcu* | This study |
